# PHANOTATE: a novel approach to gene identification in phage genomes

**DOI:** 10.1093/bioinformatics/btz265

**Published:** 2019-04-25

**Authors:** Katelyn McNair, Carol Zhou, Elizabeth A Dinsdale, Brian Souza, Robert A Edwards

**Affiliations:** 1 Computational Sciences Research Center, San Diego State University, San Diego, CA 92182, USA; 2 Lawrence Livermore National Laboratory, Livermore, CA 94550, USA; 3 Department of Biology, San Diego State University, San Diego, CA 92182, USA; 4 Viral Information Institute, San Diego State University, San Diego, CA 92182, USA

## Abstract

**Motivation:**

Currently there are no tools specifically designed for annotating genes in phages. Several tools are available that have been adapted to run on phage genomes, but due to their underlying design, they are unable to capture the full complexity of phage genomes. Phages have adapted their genomes to be extremely compact, having adjacent genes that overlap and genes completely inside of other longer genes. This non-delineated genome structure makes it difficult for gene prediction using the currently available gene annotators. Here we present PHANOTATE, a novel method for gene calling specifically designed for phage genomes. Although the compact nature of genes in phages is a problem for current gene annotators, we exploit this property by treating a phage genome as a network of paths: where open reading frames are favorable, and overlaps and gaps are less favorable, but still possible. We represent this network of connections as a weighted graph, and use dynamic programing to find the optimal path.

**Results:**

We compare PHANOTATE to other gene callers by annotating a set of 2133 complete phage genomes from GenBank, using PHANOTATE and the three most popular gene callers. We found that the four programs agree on 82% of the total predicted genes, with PHANOTATE predicting more genes than the other three. We searched for these extra genes in both GenBank’s non-redundant protein database and all of the metagenomes in the sequence read archive, and found that they are present at levels that suggest that these are functional protein-coding genes.

**Availability and implementation:**

https://github.com/deprekate/PHANOTATE

**Supplementary information:**

Supplementary data are available at *Bioinformatics* online.

## Introduction

Phages, viruses that infect bacteria, provide unique challenges for bioinformatics. There is a limit to how much DNA can be packaged in a capsid, and therefore phage genomes are generally short, typically in the range 20–100 kb. By necessity, their genomes are compact: phage genes are shorter than their bacterial homologs are frequently co-transcribed, and adjacent open reading frames (ORFs) often overlap (Kang *et al.*, 2017). In a few cases, phage genes are encoded within each other (Cahill *et al.*, 2017; Summer *et al.*, 2007). In contrast, bacterial genes generally are longer, separated by intergenic spacers and frequently switch strands (Kang *et al.*, 2017). There are no bioinformatics tools specifically designed to identify genes in phage genomes, so algorithms designed to identify bacterial genes are typically used (McNair *et al.*, 2018). For example, from 31 phage genomes published between October 14, 2016 and August 1, 2018, the genes in ten phage genomes were identified by GeneMark software (GeneMark/GeneMarkS/GeneMark.hmm), the genes in 10 phage genomes were identified by RAST, the genes in 7 phage genomes by Glimmer, 3 phage genomes each by Geneious, the NCBI ORF Finder, PHAST (which uses Glimmer as a gene caller; Arndt *et al.*, 2016), PROKKA (which uses Prodigal as a default gene caller; Seemann, 2014), 2 phage genomes by Prodigal and 1 phage genome by MetaVir, RASTtk, SerialCloner or SnapGene (Supplementary Table S1; note that in many publications several different tools were used to identify genes in phage genomes). Each of these algorithms relies on information that is not available and calculations that are not possible with short genomes. For example, there are no conserved genes in phage genomes that can be used to build universal training sets (Rohwer and Edwards, 2002), fewer genes means the statistics used to identify start codons are less accurate (Wu *et al.*, 2003), and because many phage genes or the proteins they encode have no homolog in the databases, similarity searches are unreliable (Roux *et al.*, 2015). There are alternate gene calling approaches, such as using positional nucleotide frequency (Besemer and Borodovsky, 1999), or the multivariate entropy of amino acid usage used by Glimmer (Ouyang *et al.*, 2004), but these are designed for complete bacterial genomes and have not been optimized for use with phage genomes.

Here, we introduce a novel method for gene identification that is specifically designed for phage genomes. We make several presumptions based on studying hundreds of phages genomes. First, we noted that since phages have physical limits on their genome sizes they contain minimal non-coding DNA. Second, we showed that phage genes are usually on the same strand of the DNA, presumably because they are co-transcribed (Akhter, 2012; Kang *et al.*, 2017). Based on these observations, we designed a completely novel approach to phage gene identification, tiling opening reading frames to minimize non-coding DNA bases and strand switching. We treat a phage genome as a network of paths in which ORFs are more favorable, and overlaps and gaps are less favorable. We solved this weighted graph problem using the Bellman-Ford algorithm (Bellman, 1958; Ford, 1956), and by optimizing the parameters for phages genomes we are able to enhance phage gene prediction algorithms. In the absence of supporting data to confirm our new predictions, we turned to high-volume sequence similarity searches to explore the predicted proteins. Regions of the genome that encode proteins are more likely to be conserved at the amino acid level than regions that encode regulatory regions, replication regions, sites of integration and other, DNA-based, information components of the phage genome (Badger and Olsen, 1999). These searches showed that the predicted phage genes might encode novel proteins that have been missed by existing gene callers designed to annotate bacterial genomes.

## 2 Materials and methods

### 2.1 The PHANOTATE algorithm

The first step PHANOTATE takes in identifying the genes in a phage genome is creating a weighted graph from the ORFs in that genome. By default, we allow for three start codons (*codons_start_ = {ATG, GTG, TTG*}), and three stop codons (*codons_stop_ = {TAA, TAG, TGA}*), and the default minimum length of an ORF is 90 nt. The directed weighted graph consists of nodes that represent start and stop codons, and edges that represent either an *ORF* if the edge connects a start codon to a subsequent stop codon in the same reading frame; a *gap* if the edge connects a stop codon to a subsequent start codon in any reading frame on the same strand, or if the edge connects a stop codon to a subsequent stop codon on the alternate strand; or an *overlap* if the edge connects a stop codon to a preceding start codon in any other reading frame on the same strand, or to a preceding stop codon on the alternate strand. Since phages rarely have >300 bp of untranslated DNA, and to reduce computational burden, we only connect ORFs within ±300 bp of each other. When there is a very large span without an ORF, we connect ORFs on each side of the region with a linear penalty.

For each edge, we calculate a weight depending on the feature type: ORF, overlap, or gap. To calculate the weight of an ORF (*w*_orf_*)*, we use an adjusted likelihood of not finding a stop codon in an ORF of this length. We count the fraction of each base in each ORF, and use that to determine the overall probability encountering a stop codon over the entire ORF:

(1) P(stop) =P(TAA)+P(TAG)+P(TGA)

We then calculate *P*(not stop) to obtain the probability of NOT encountering a stop codon:

(2) P(not stop)=1-P(stop)

Using *P*(*not stop*) alone to calculate the path through the genome is sufficient for genomes with an average GC content; however high GC content genomes have extremely long, spurious, ORFs caused by their bias of generally having a G or C in the third codon position of their protein-encoding genes, which then forces a C or G in the first position in the opposite strand, limiting the options for including stop codons in the genome. To overcome this we incorporated two GC frame plot scores into our final calculation. The initial GC frame plot (GCFP) score was inspired by Prodigal, but we have adapted that and we also include both minimum GC frame plot and maximum GC frame plot. We start by reading the three frames of the genome one base at a time, looking at the codon starting at that base, and calculating the %GC content over a 120 bp window for each of the three reading frames. Taking the set of ORFs that start with ATG, we iterate through the codons of those ORFs and determine which position (first, second or third) has the maximum GC content, and maintain a running total for that position. Similarly, we calculate a GC frame plot minimum score by recording the minimum GC content (pseudocode is provided in Supplementary Material). This gives us a count of the frequency of the three positions in all ORFs that start with ATG and can be used to estimate the preferred reading frame at any location. We translate these three numbers into scores by dividing each by the counts for the position with the highest count, bringing the preferred maximum GC position to 1, and the others to <1. This yields a set of three position scores that range between 0 and 1, with 1 being the maximal or minimal GC frame. For instance, if the input genome had a bias where half of its max GC frame was in the third frame, and the other half split evenly between the first and second frame, once normalized, the GCFPmax scores would be [0.5, 0.5, 1]. The GC frame plot scores are used to exponentiate the *P*(not stop) score. For example, if a codon’s GCFPmax score was 1, which would match the preferred frame, then *P*(not stop) is unchanged. However, if a codon’s GCFPmax score is less than 1, indicating that the current ORF is in a different frame to the preferred GC frame at that location in the genome, then that codon’s *P*(not stop) value is reduced in the final calculation.

Scores for ORFs are modified by a weighted *ribosomal-binding site* (RBS) score. Since little is currently known about the diversity of RBSs in phages, we employed a similar likelihood-based Shine-Dalgarno RBS system used previously (Hyatt *et al.*, 2010). We plan to add a more rigorous non-Shine-Dalgarno RBS motif finder in subsequent versions of PHANOTATE. In addition, we adjust the ORF score based on the likelihood that the first codon is a start. We created a normalized frequency of start codons based on all genes predicted in GenBank in 2133 phage genomes. Finally, the weight is negated to denote these edges as favorable in the network.

The calculation to generate a weighted score *w*_orf_ for each ORF in the graph is shown in Equation (5).

When continuing from a stop codon either in a gap or an overlap, the next ORF maybe on either strand of the DNA sequence. However, phage genes are usually on the same strand, and unlike bacterial genes, they rarely switch strands (Kang *et al.*, 2017). If a strand switch occurs, then a strand switch penalty is included in the weight of the gap or overlap, where *P*(switch) is equal to 0.05, otherwise no penalty is added: *P*(switch) = {0, 0.05}. This penalty is the multiplicative inverse of the probability of a strand switch occurring, which was calculated from our set of annotated genes derived from the 2133 phage genomes to occur at a rate of ∼5% per protein-encoding gene (in contrast, the rate per bacterial protein-encoding gene is ∼25%).

Since gap weights (*w*_gap_) need to be proportionally scaled to ORF weights, we use a similar weight as ORFs (*w*_orf_). They are not corrected for GC frame plot, and use a genome-wide average probability of not finding a stop codon $\underline{P}$ (not stop) that is exponentiated by the length of the gap, and then the positive multiplicative inverse is taken and combined with *P*(switch) (Equation 3).

(3) $$w_{\mathrm{gap}}=\frac{1}{\left(\underline{P}(\mathrm{not}\;\mathrm{stop}{)}^{\mathrm{len}}\right)}+\frac{1}{P(\mathrm{switch})}\;$$

Overlap weights (*w*_overlap_) also need to be proportionally scaled to ORF weights, so they are calculated by finding the average of the two coding weights of the ORFs in the overlap, and then exponentiating by the length, *n*, of the overlap (Equation 4). If a strand switch occurs, then a penalty is added to the gap weight as noted above.

(4) $$w_{\mathrm{overlap}}=\frac{1}{(\frac{P(\mathrm{not}\;\mathrm{stop}{)}_{1}+\;P(\mathrm{not}\;\mathrm{stop}{)}_{2}}{2}{)}^{\mathrm{len}}}+\frac{1}{P(\mathrm{switch})}$$

In order to use these weights in with the Bellman-Ford algorithm, they must be transformed into ‘distances’, so for each of the above weights, we take the multiplicative inverse of the probabilities to create a weighted graph network. Our novel C-based implementation of the Bellman-Ford algorithm is then used to find the shortest path through the network.

### 2.2 Comparison with other gene callers

We compared gene identification between PHANOTATE and the three most popular gene callers used to identify genes in phages (Supplementary Table S1): GeneMarkS, Glimmer, and Prodigal using a set of 2133 complete phage genomes, which were downloaded from the GenBank FTP server (Benson *et al.*, 2017). We did not include nine *Mycoplasma* and *Spiroplasma* phages, which use an alternative genetic code. We ran PHANOTATE and each of the three alternative gene callers with default (or ‘phage’ if available) parameters on each phage genome, as is done for most phage genome annotation projects (Supplementary Table S1). In addition, the ‘meta’ option was used to allow Prodigal to run on genomes smaller than 20 kb.

(5) $$w_{\mathrm{orf}}=-\frac{1}{\prod_{c=1}^{\mathrm{codons}}(P(\mathrm{not}\;\mathrm{stop}{)}^{\mathrm{GCFPma}x_{\mathrm{maxGCframe}\left(c\right)}\mathrm{GCFPmi}n_{\mathrm{minGCframe}\left(c\right)}})}*\mathrm{RBS}*\mathrm{START}$$

To mask out functional, but non-protein-coding regions of the genomes, we used the program tRNAscan-SE to find the tRNA genes in each genome. To compare the algorithms, we counted the number of ORFs predicted by each respective algorithm and compared those predictions to the corresponding genes in GenBank.

### 2.3 Statistical analyses

All analyses were performed in Python using the statsmodels and scipy modules (scipy.org) (Jones *et al.*, 2001; Seabold and Perktold, 2010). ANOVA, Tukey’s honest significant difference test, Levene’s test, Cohen’s *f*^2^ test and *t*-tests were performed on ln(*x* + 1)-normalized length or count data.

### 2.4 Validation against the sequence read archive

**Fig. 1.**
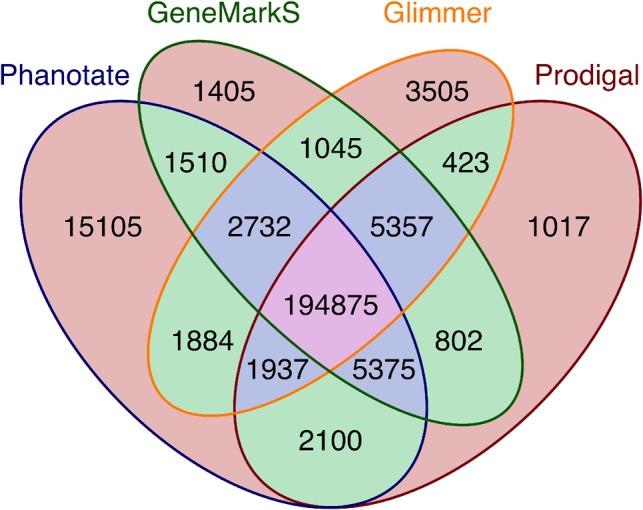
Number of genes predicted by each of four different gene prediction algorithms and the combinations thereof. Orange background: predicted by a single algorithm; green background: predicted by two algorithms; blue background: predicted by three algorithms; pink background: predicted by all four algorithms

In the absence of direct protein measurements, we used conserved similarity to test whether ORFs are likely to encode proteins. To create a positive control set, we combined the 223 385 ORFs that were predicted to encode proteins by one or more of Glimmer, GeneMarkS or Prodigal. To create a negative control set, we identified the 1 122 336 ORFs over 90 nt that were *not* predicted to encode proteins by any software (Glimmer, GeneMarkS, Prodigal or PHANOTATE). Finally, we also identified the 15 105 ORFs that were unique to PHANOTATE (Fig. 1). We previously developed partie (Torres *et al.*, 2017) to identify the random community genomes (metagenomes) in the NCBI Sequence Read Archive (SRA) (NCBI Resource Coordinators, 2016). We used lastal (Kiełbasa *et al.*, 2011; Sheetlin *et al.*, 2014) to compare six-frame translations of a 100 000 read sample of the sequence reads from these metagenomes in the SRA to the predicted protein sequences from the ORFs. Sequences with an expect value <1 × 10^−^^10^ were considered significant. The differences in means were compared using a one-way ANOVA followed by a *post hoc* Tukey’s test to identify the variables driving any difference. Normality was tested using Levene’s test (Fowler *et al.*, 1998). Cohen’s *f*^2^ test was used to determine effect size.

These datasets are uneven and large and therefore direct comparisons may lead to small effects being found to be significant. To overcome this we measure both Cohen’s *f*^2^ and d values to measure effect size (Cohen, 1988; Nakagawa and Cuthill, 2007). In addition, we subsample 1000 proteins with replacement at random from the entire pool of ORFs and use those in the ANOVA. We repeat this calculation 1000 times to determine whether the PHANOTATE predictions are similar to either the set of positive predicted proteins or the negative control set of ORFs that were not predicted to encode proteins.

The Git repository contains a detailed description of the approach used to compare the SRA reads to the predicted ORFs, contains a link to the alignment data, and contains Jupyter notebooks with the statistical analysis reported below.

https://github.com/deprekate/PHANOTATE

## 3 Results

PHANOTATE is a novel gene caller designed explicitly to identify phage genes. We used the Bellman-Ford algorithm to treat the genome like a path, and parameterized the search by calculating the weights from 2133 phage genomes in GenBank. To test PHANOTATE, we calculated the number of genes predicted by our algorithm and compared that to the genes predicted by those algorithms typically used to call genes in phages (Supplementary Table S1), namely Glimmer (Ouyang *et al.*, 2004), GeneMarkS (Besemer and Borodovsky, 1999) and Prodigal (Hyatt *et al.*, 2010). In total, we identified 239 072 genes from 2133 phage genomes (Table 1).

**Table 1.** Numbers and lengths of the genes predicted by the different gene callers

Gene caller	Number of genes	Mean length (nt)	SD of gene length (nt)
PHANOTATE	225 518	603	708
GeneMarkS	213 101	628	719
Glimmer	211 278	631	719
Prodigal	211 886	631	720

There was no statistically significant difference in the mean lengths of the genes predicted Glimmer or Prodigal, while the mean lengths of the genes predicted by PHANOTATE and GeneMarkS were statistically significantly different to those called by the other algorithms [*F*(3, 861 779) = 440.45, *P* = 0.0]. However, the effect size of the difference was very small (*d* < 0.1 in every pairwise comparison).

The Jaccard index (*J*) calculated from these results show that Prodigal and GeneMarkS are the most similar in their predictions (*J(Prodigal, GeneMarkS)* = 0.94); Glimmer is similar to both Prodigal and GeneMarkS (*J(Glimmer, Prodigal)* = *J(Glimmer, GeneMarkS)* = 0.92); while PHANOTATE is the most different because of the large number of ORFs that it predicts as proteins that the others do not (see below; *J(PHANOTATE, Prodigal)*) = 0.88; *J(PHANOTATE, Glimmer)* = *J(PHANOTATE, GeneMarkS)* = 087).

Each of the tools identified a set of predicted genes that were not identified by any of the other software. PHANOTATE version 1.0 predicted 15 105 genes (6% of the total number of genes predicted by all software) that were not predicted by other gene prediction algorithms. An ANOVA comparison between the lengths of the genes identified by 1, 2, 3 or 4 gene callers identified significant variation [*F*(1, 861 781) = 21 312.85, *P *=* *0.0], but the effect size was very small (*d* = 0.02). A *post hoc* Tukey’s test showed that there was no difference between the lengths of genes identified by a single gene caller or two gene callers (*P* > 0.05), but that all other pairwise comparisons were different. When we consider just the unique genes that were identified by each algorithm the ANOVA comparison identified significant variation in the lengths of the genes [*F*(3, 20 856) = 56.6, *P *=* *0], but again the effect size was very small (*d* = 0.01). The *post hoc* Tukey’s test showed that there were two groups that were significantly different between groups but not within groups (*P *<* *0.05). Glimmer (*M *=* *217 nt, SD = 174.35) and Prodigal (*M *=* *226 nt, SD = 151.58) had indistinguishable mean lengths of unique genes, while the mean lengths of PHANOTATE (*M *=* *210 nt, SD = 245.94) and GeneMarkS (*M *=* *183 nt, SD = 109.06) were indistinguishable.

We cannot simply rely on the GenBank annotations to be correct. First, the proteins annotated in GenBank are typically predicted by the gene callers used in this comparison (Supplementary Table S1). Second, many of the current phage genome annotations in GenBank are filled with false positives. For example, in the Shiga toxin-converting phages (NC_004913 and NC_004914), every ORF longer than 160 bp has been annotated as a protein-coding gene. There are also abundant examples of false negatives, protein-coding genes present in the genome that were not identified by the annotation software. The most obvious false negatives are genes shorter than 100 bp, since this is an often-used arbitrary minimum cutoff. Small genes that do not show strong coding signals, such as shared homology to known or hypothetical genes in the databases or shared codon usage, are often excluded by other gene annotators in an effort to minimize false positives.

The best experimental approach to determine whether these genes encode proteins would be to identify the proteins via proteomics. However, there are few published phage proteomics studies (Fagerquist *et al.*, 2014; Pope *et al.*, 2014), and in those studies, the raw proteomics data are not provided. Rather the authors only indicate which ORFs were matched, frequently using proprietary software and typically using gene calls made using the algorithms discussed here. This precludes our ability to use proteomics data to validate gene identification in phages.

In the absence of third-party validation datasets and experimental datasets, we turned to evolution to test whether the genes we predict in these phages may encode proteins. We hypothesized that protein-encoding genes are more likely to be evolutionarily conserved than ORFs that are not translated into proteins. Protein-encoding genes are constrained by the function of the protein. A variant of this approach has previously been used to identify genes in bacterial genomes (Badger and Olsen, 1999). When we compared the genes that PHANOTATE predicted to the proteins in the GenBank non-redundant (nr) protein database (Benson et al., 2017), there was significant similarity to 23% of the predicted proteins (expect value < 10^−^^10^). This is similar to the 1–30% of phage proteins that typically have similarity to the GenBank nr database, and the remained is often called the ‘phage dark matter’ (Mokili *et al.*, 2012). The mean lengths of the predicted genes that did not match to GenBank (243 nt) was significantly shorter than the mean length of those genes that matched GenBank (229 nt) [*t*(1000) = 3.02, *P* < 0.005] but the effect size was small (*d* = 0.19). This may suggest that shorter proteins are under-represented in the database because of arbitrary lower limits on gene callers, shorter proteins have less statistical significance in similarity searches, or PHANOTATE is identifying more, shorter, ORFs and incorrectly suggesting they are proteins. We, therefore, sought an additional assurance of the genes predicted by PHANOTATE.

**Fig. 2.**
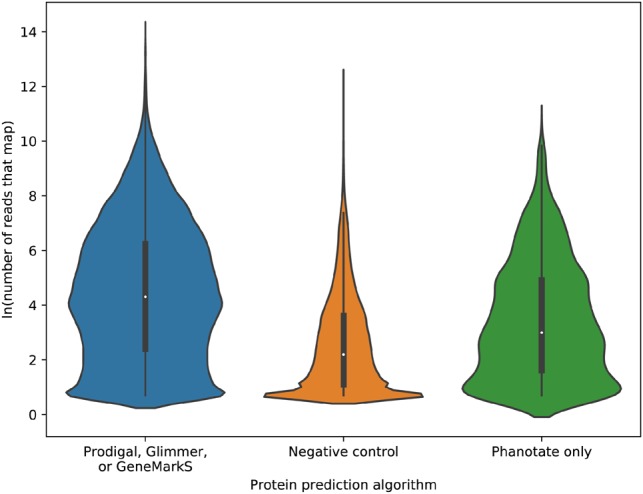
Violin plot of the ln(number or reads that map) to each of the ORFs predicted either by one (or more) of Prodigal, Glimmer or GeneMarkS; by no gene prediction algorithms (negative control); or by PHANOTATE alone

For a more rigorous analysis of the ability of sequence similarity to discriminate between coding and non-coding genes, we turned to the largest repository of sequence data, the NCBI SRA (NCBI Resource Coordinators, 2016). Specifically, we extracted 94 652 random community metagenomes we previously identified (Torres *et al.*, 2017). We constructed two control datasets: a set of presumed positive predictions comprised of all ORFs predicted by Glimmer, GeneMarkS and/or Prodigal (but not those only predicted by PHANOTATE), and a set of known negative annotations of ORFs that are longer than 90 bp and not predicted to encode proteins by any of the software used here, including PHANOTATE. We mapped the reads from the SRA to the ORFs using the translated search algorithm lastal (Kiełbasa *et al.*, 2011; Sheetlin *et al.*, 2014). When we compared the number of reads that mapped for all ORFs that had at least one read map, significantly more reads mapped to the ORFs predicted to be proteins (mean 1871.5 reads mapped; standard deviation 15 933.2), than our negative control set (mean 136.0 reads mapped; standard deviation 1316.9) (Fig. 2) [*F*(2, 149 770) = 37 900, *P *=* *0.00]. There was a large effect size for this comparison (*d* = 0.9), as can be seen in Figure 2. This analysis confirms that we are more likely to find reads mapping to ORFs if they encode proteins than if they do not encode proteins, and therefore we can use this approach to determine whether the ORFs predicted by PHANOTATE alone are likely to encode proteins.

When we compare the ORFs that are only predicted by PHANOTATE and not predicted by the other ORF callers (∼6% of all the ORFs identified) with the two control sets, 72% of the time the ORFs predicted by PHANOTATE had mean read abundance that was indistinguishable from the mean abundance of the true proteins, but 79% of the time the mean read abundance was similar to the ORFs that were not predicted to be proteins. Similarly, the medium effect size suggests that similarities to ORFs identified by PHANOTATE lie between those predicted by any gene caller (*d* = 0.42) and those not predicted by any caller (*d* = 0.47) as can be seen in Figure 2. The PHANOTATE predictions, therefore, lie between the ‘true positives’ from the other software and the ‘true negatives’ of all other ORFs, suggesting, but not confirming that they may encode real proteins.

One of the unique features of PHANOTATE is that it is essentially reference free. Other programs, such as Prodigal, GeneMark and Glimmer, use hidden Markov models that require either a priori knowledge of the composition of protein-encoding genes or the identification of sufficient protein-encoding genes in the genome to build a training set. This is problematic when annotating phage genomes since most potential ORFs do not have homology to any known gene and the small phage genomes do not provide enough candidates to create a robust training set. In addition, many phage genes are horizontally transferred, and thus have different properties and signals from each other. Future versions of PHANOTATE will include the option to use these various gene properties, including hexamer frequency, codon bias and non-Shine-Dalgarno RBS detection, and will also provide a mechanism to mask functional noncoding bases, such as those in RNAs, repeats, and *att* sites to further increase the accuracy of the gene calls.

## Funding

This work has been supported by the US Department of Defense: Defense Threat Reduction Agency grant number [DTRA10027-20149] and by a STEM scholarship award funded by the National Science Foundation grant [DUE-1259951] and the Computational Science Research Center at SDSU.

*Conflict of Interest*: None declared.

## Supplementary Material

btz265_Supplementary_Data
