# Supplementary material for: PHANOTATE: a novel approach to gene identification in phage genomes
Source: Bioinformatics. 2019 Apr 25;35(22):4537–42. doi: 10.1093/bioinformatics/btz265 (PMC6853651; doi:10.1093/bioinformatics/btz265)
Supplement: btz265_Supplementary_Data [file btz265_supplementary_data.zip › btz265-suppl_data/pseudocode.docx]

Maxframe

max_frames = []

window = 120

FOR i = 1 to length(nucleotides)

position_counts = [0,0,0]

j = 0

WHILE j < window

IF nucleotides[i+j] == “C” || nucleotides[i+j] == “G”

position_counts[1] = position_counts[1] + 1

ENDIF

IF nucleotides[i+j+1] == “C” || nucleotides[i+j+1] == “G”

position_counts[2] = position_counts[2] + 1

ENDIF

IF nucleotides[i+j+2] == “C” || nucleotides[i+j+2] == “G”

position_counts[3] = position_counts[3] + 1

ENDIF

j <- j + 3

ENDWHILE

max_frames[i] = max_index(position_counts)

ENDFOR

max_frames = max_frames[ window/2 : length(nucleotides) ]

RETURN max_frames

GCFPmax

max_gc_frame = Maxframe

frame_counts = [0,0,0]

FOR orf in orfs_startwith_atg

FOR codon in codons(orf)

i = max_gc_frame(codon)

frame_counts[i] = frame_counts[i] +1

ENDFOR

ENDFOR

FOR i = 1 to 3

frame_counts[i] = frame_counts[i] / max(frame_counts)

ENDFOR

RETURN frame_counts
