# Supplementary material for: PHANOTATE: a novel approach to gene identification in phage genomes
Source: Bioinformatics. 2019 Apr 25;35(22):4537–42. doi: 10.1093/bioinformatics/btz265 (PMC6853651; doi:10.1093/bioinformatics/btz265)
Supplement: btz265_Supplementary_Data [file btz265_supplementary_data.zip › btz265-suppl_data/SupplementalTable1.pdf]

Supplemental Table 1. Gene Callers Used in Phage Genome Annotation Papers from 10/14/2016 through 8/1/2018

Paper	Date Published	Gene caller[1,2,3]	Reference
Complete nucleotide sequence of a new filamentous phage, Xf109, which integrates its genome into the chromosomal DNA of Xanthomonas oryzae	10/14/2016	SerialCloner	(Yeh, 2017)
Complete Genome Sequences of 38 Gordonia sp. Bacteriophages	1/5/2017	GeneMark; Glimmer	(Pope *et al.*, 2017)
Large Preferred Region for Packaging of Bacterial DNA by phiC725A, a Novel Pseudomonas aeruginosa F116-Like Bacteriophage	1/6/2017	Geneious	(Pourcel *et al.*, 2017)
Things Are Getting Hairy: Enterobacteria Bacteriophage vB_PcaM_CBB	1/24/2017	Glimmer; GeneMarkS	(Buttimer *et al.*, 2017)
Genome Analysis of a Novel Broad Host Range Proteobacteria Phage Isolated from a Bioreactor Treating Industrial Wastewater	1/28/2017	Glimmer	(de Leeuw *et al.*, 2017)
Complete genome sequence of Pseudoalteromonas phage vB_PspS-H40/1 (formerly H40/1) that infects Pseudoalteromonas sp. strain H40 and is used as biological tracer in hydrological transport studies	2/2/2017	RAST; Glimmer; GeneMark.hmm	(Kallies *et al.*, 2017)
Genome of a giant bacteriophage from a decaying Trichodesmium bloom	2/22/2017	RAST	(Pfreundt *et al.*, 2017)
Phage-host interactions in Streptococcus thermophilus: Genome analysis of phages isolated in Uruguay and ectopic spacer acquisition in CRISPR array	3/6/2017	NCBI ORF finder	(Achigar *et al.*, 2017)
Discovery and Complete Genome Sequence of a Bacteriophage from an Obligate Intracellular Symbiont of a Cellulolytic Protist in the Termite Gut	3/17/2017	RAST; METAVIR	(Pramono *et al.*, 2017)
Genome Sequence of Escherichia coli Tailed Phage Utah	3/30/2017	Geneious	(Leavitt *et al.*, 2017)
Genomic Diversity of Type B3 Bacteriophages of Caulobacter crescentus	4/10/2017	RAST	(Ash *et al.*, 2017)
Isolation and Complete Genome Sequence	4/20/2017	RAST	(Gong *et al.*, 2017)

of a Novel Pseudoalteromonas Phage PH357 from the Yangtze River Estuary			
Genome Sequence of Serratia marcescens Phage BF	6/8/2017	Prodigal	(Casey *et al.*, 2017)
Characterization and Complete Genome Sequence of a Novel Siphoviridae Bacteriophage BS5	7/1/2017	RAST	(Meng *et al.*, 2017)
Complete Genome Sequence of Streptococcus pneumoniae Virulent Phage MS1	7/13/2017	RASTtk	(Kot *et al.*, 2017)
Biology and Genomics of an Historic Therapeutic Escherichia coli Bacteriophage Collection	8/30/2017	PROKKA	(Baig *et al.*, 2017)
Genome characteristics and environmental distribution of the first phage that infects the LD28 clade, a freshwater methylotrophic bacterial group	9/19/2017	RAST; Glimmer; GeneMark	(Moon *et al.*, 2017)
Genome Sequences of Chancellor, Mitti, and Wintermute, Three Subcluster K4 Phages Isolated Using Mycobacterium smegmatis mc2155	11/9/2017	Glimmer; GeneMark	(Edgington *et al.*, 2017)
Structure, proteome and genome of Sinorhizobium meliloti phage ΦM5: A virus with LUZ24-like morphology and a highly mosaic genome	12/3/2017	GeneMark.hmm; NCBI ORF Finder	(Johnson *et al.*, 2017)
Complete genome sequence of lytic bacteriophage RG-2014 that infects the multidrug resistant bacterium Delftia tsuruhatensis ARB-1	12/18/2017	GeneMarkS	(Bhattacharjee *et al.*, 2017)
Genomic analysis of WCP30 Phage of Weissella cibaria for Dairy Fermented Foods	12/31/2017	NCBI ORF finder	(Lee and Park, 2017)
The genome sequence of Escherichia coli tailed phage D6 and the diversity of Enterobacteriales circular plasmid prophages	1/2/2018	Snapgene; GeneMarkS	(Gilcrease and Casjens, 2018)
The Novel Phages phiCD5763 and phiCD2955 Represent Two Groups of Big Plasmidial Siphoviridae Phages of Clostridium difficile	1/22/2018	PROKKA	(Ramírez-Vargas *et al.*, 2018)
Complete Genome Sequence of EtG, the First Phage Sequenced from Erwinia tracheiphila	2/22/2018	RAST; PHAST	(Andrade-Domínguez *et al.*, 2018)

Comparative genomics of Cp8viruses with special reference to Campylobacter phage vB_CjeM_los1, isolated from a slaughterhouse in Ireland	4/23/2018	Glimmer; Prodigal	(O'Sullivan *et al.*, 2018)
Rates of Mutation and Recombination in Siphoviridae Phage Genome Evolution over Three Decades	5/1/2018	RAST	(Kupczok *et al.*, 2018)
Complete Genome Sequence of Sinorhizobium meliloti Bacteriophage HMSP1-Susan	5/3/2018	GeneMark	(Fleagle *et al.*, 2018)
Genome Sequence of a New Siphoviridae Phage Found in a Brazilian Bacillus thuringiensis Serovar israelensis Strain	5/31/2018	Geneious	(Campos *et al.*, 2018)
Genome Sequence of the Pseudomonas protegens Phage ΦGP100	6/21/2018	RAST; PHAST	(Vacheron *et al.*, 2018)
Complete Genome Sequence of Escherichia coli Phage vB_EcoS Sa179lw, Isolated from Surface Water in a Produce-Growing Area in Northern California	7/5/2018	PROKKA	(Liao *et al.*, 2018)
Sequencing, genome analysis and host range of a novel Ralstonia phage, RsoP1EGY, isolated in Egypt	8/1/2018	PHAST; GeneMark	(Ahmad *et al.*, 2018)

Footnotes:

[1]PHAST uses Glimmer as the default gene caller (Zhou *et al.*, 2011)

[2]PROKKA uses Prodigal as the default gene caller (Seemann, 2014)

[3]RAST uses the RAST gene caller by default, but Glimmer and Prodigal can be specified as alternate gene callers (McNair *et al.*, 2018)

References

Achigar,R. *et al.* (2017) Phage-host interactions in Streptococcus thermophilus: Genome analysis of phages isolated in Uruguay and ectopic spacer acquisition in CRISPR array. *Sci. Rep.*, **7**, 43438.

Ahmad,A.A. *et al.* (2018) Sequencing, genome analysis and host range of a novel Ralstonia phage, RsoP1EGY, isolated in Egypt. *Arch. Virol.*, **163**, 2271–2274.

Andrade-Domínguez,A. *et al.* (2018) Complete Genome Sequence of EtG, the First Phage Sequenced from Erwinia tracheiphila. *Genome Announc.*, **6**.

Ash,K.T. *et al.* (2017) Genomic Diversity of Type B3 Bacteriophages of Caulobacter crescentus. *Curr. Microbiol.*, **74**, 779–786.

Baig,A. *et al.* (2017) Biology and Genomics of an Historic Therapeutic Escherichia coli Bacteriophage Collection. *Front. Microbiol.*, **8**, 1652.

Bhattacharjee,A.S. *et al.* (2017) Complete genome sequence of lytic bacteriophage RG-2014

that infects the multidrug resistant bacterium Delftia tsuruhatensis ARB-1. *Stand. Genomic Sci.*, **12**, 82.
Buttimer,C. *et al.* (2017) Things Are Getting Hairy: Enterobacteria Bacteriophage vB_PcaM_CBB. *Front. Microbiol.*, **8**, 44.
Campos,F.S. *et al.* (2018) Genome Sequence of a New Siphoviridae Phage Found in a Brazilian Bacillus thuringiensis Serovar israelensis Strain. *Genome Announc.*, **6**.
Casey,E. *et al.* (2017) Genome Sequence of Serratia marcescens Phage BF. *Genome Announc.*, **5**.
Edgington,N.P. *et al.* (2017) Genome Sequences of Chancellor, Mitti, and Wintermute, Three Subcluster K4 Phages Isolated Using Mycobacterium smegmatis mc2155. *Genome Announc.*, **5**.
Fleagle,B. *et al.* (2018) Complete Genome Sequence of Sinorhizobium meliloti Bacteriophage HMSP1-Susan. *Genome Announc.*, **6**.
Gilcrease,E.B. and Casjens,S.R. (2018) The genome sequence of Escherichia coli tailed phage D6 and the diversity of Enterobacteriales circular plasmid prophages. *Virology*, **515**, 203–214.
Gong,Z. *et al.* (2017) Isolation and Complete Genome Sequence of a Novel Pseudoalteromonas Phage PH357 from the Yangtze River Estuary. *Curr. Microbiol.*, **74**, 832–839.
Johnson,M.C. *et al.* (2017) Structure, proteome and genome of Sinorhizobium meliloti phage ΦM5: A virus with LUZ24-like morphology and a highly mosaic genome. *J. Struct. Biol.*, **200**, 343–359.
Kallies,R. *et al.* (2017) Complete genome sequence of Pseudoalteromonas phage vB_PspS-H40/1 (formerly H40/1) that infects Pseudoalteromonas sp. strain H40 and is used as biological tracer in hydrological transport studies. *Stand. Genomic Sci.*, **12**, 20.
Kot,W. *et al.* (2017) Complete Genome Sequence of Streptococcus pneumoniae Virulent Phage MS1. *Genome Announc.*, **5**.
Kupczok,A. *et al.* (2018) Rates of Mutation and Recombination in Siphoviridae Phage Genome Evolution over Three Decades. *Mol. Biol. Evol.*, **35**, 1147–1159.
Leavitt,J.C. *et al.* (2017) Genome Sequence of Escherichia coli Tailed Phage Utah. *Genome Announc.*, **5**.
de Leeuw,M. *et al.* (2017) Genome Analysis of a Novel Broad Host Range Proteobacteria Phage Isolated from a Bioreactor Treating Industrial Wastewater. *Genes* , **8**.
Lee,Y.-D. and Park,J.-H. (2017) Genomic analysis of WCP30 Phage of Weissella cibaria for Dairy Fermented Foods. *Korean J Food Sci Anim Resour*, **37**, 884–888.
Liao,Y.-T. *et al.* (2018) Complete Genome Sequence of Escherichia coli Phage vB_EcoS Sa179lw, Isolated from Surface Water in a Produce-Growing Area in Northern California. *Genome Announc.*, **6**.
McNair,K. *et al.* (2018) Phage Genome Annotation Using the RAST Pipeline. In, Clokie,M.R.J. *et al.* (eds), *Bacteriophages: Methods and Protocols, Volume 3.* Springer New York, New York, NY, pp. 231–238.
Meng,X. *et al.* (2017) Characterization and Complete Genome Sequence of a Novel Siphoviridae Bacteriophage BS5. *Curr. Microbiol.*, **74**, 815–820.
Moon,K. *et al.* (2017) Genome characteristics and environmental distribution of the first phage that infects the LD28 clade, a freshwater methylotrophic bacterial group. *Environ. Microbiol.*, **19**, 4714–4727.
O'Sullivan,L. *et al.* (2018) Comparative genomics of Cp8viruses with special reference to Campylobacter phage vB_CjeM_los1, isolated from a slaughterhouse in Ireland. *Arch.*

Virol., **163**, 2139–2154.
Pfreundt,U. *et al.* (2017) Genome of a giant bacteriophage from a decaying Trichodesmium bloom. *Mar. Genomics*, **33**, 21–25.
Pope,W.H. *et al.* (2017) Complete Genome Sequences of 38 Gordonia sp. Bacteriophages. *Genome Announc.*, **5**.
Pourcel,C. *et al.* (2017) Large Preferred Region for Packaging of Bacterial DNA by phiC725A, a Novel Pseudomonas aeruginosa F116-Like Bacteriophage. *PLoS One*, **12**, e0169684.
Pramono,A.K. *et al.* (2017) Discovery and Complete Genome Sequence of a Bacteriophage from an Obligate Intracellular Symbiont of a Cellulolytic Protist in the Termite Gut. *Microbes Environ.*, **32**, 112–117.
Ramírez-Vargas,G. *et al.* (2018) The Novel Phages phiCD5763 and phiCD2955 Represent Two Groups of Big Plasmidial Siphoviridae Phages of Clostridium difficile. *Front. Microbiol.*, **9**, 26.
Seemann,T. (2014) Prokka: rapid prokaryotic genome annotation. *Bioinformatics*, **30**, 2068–2069.
Vacheron,J. *et al.* (2018) Genome Sequence of the Pseudomonas protegens Phage ΦGP100. *Genome Announc.*, **6**.
Yeh,T.Y. (2017) Complete nucleotide sequence of a new filamentous phage, Xf109, which integrates its genome into the chromosomal DNA of Xanthomonas oryzae. *Arch. Virol.*, **162**, 567–572.
Zhou,Y. *et al.* (2011) PHAST: A Fast Phage Search Tool. *Nucleic Acids Res.*, **39**, W347–W352.
